# Supplementary material for: Hot spot prediction in protein-protein interactions by an ensemble system
Source: BMC Syst Biol. 2018 Dec 31;12(Suppl 9):132. doi: 10.1186/s12918-018-0665-8 (PMC6311905; doi:10.1186/s12918-018-0665-8)
Supplement: Supplementary file 2 — All relevant MATLAB code. A simple Matlab implement of our predictor. (ZIP 19,742 kb) [file 12918_2018_665_MOESM2_ESM.zip › Additional file 2/Read ASA/ASA website/SKEMPI/2I9BE.htm]

 NetSurfP - Protein Surface Accessibility and Secondary Structure Predictions

  

|  |  |  |
| --- | --- | --- |
|  |  | NetSurfP - Protein Surface Accessibility and Secondary Structure Predictions  Technical University of Denmark |

  


---

```
# For publication of results, please cite:
# A generic method for assignment of reliability scores applied to solvent accessibility predictions.
# Bent Petersen, Thomas Nordahl Petersen, Pernille Andersen, Morten Nielsen and Claus Lundegaard
# BMC Structural Biology 2009, 9:51 doi:10.1186/1472-6807-9-51
#
# Column 1: Class assignment - B for buried or E for Exposed - Threshold: 25% exposure, but not based on RSA
# Column 2: Amino acid
# Column 3: Sequence name
# Column 4: Amino acid number
# Column 5: Relative Surface Accessibility - RSA
# Column 6: Absolute Surface Accessibility
# Column 7: Z-fit score for RSA prediction
# Column 8: Probability for Alpha-Helix
# Column 9: Probability for Beta-strand
# Column 10: Probability for Coil
E S  2I9BE                  1    0.906 106.218   0.418   0.003   0.003   0.994
B L  2I9BE                  2    0.251  45.995  -1.609   0.004   0.197   0.799
E R  2I9BE                  3    0.490 112.279   1.084   0.004   0.420   0.576
B C  2I9BE                  4    0.168  23.531  -1.953   0.004   0.616   0.381
E M  2I9BE                  5    0.321  64.272   0.190   0.021   0.756   0.223
E Q  2I9BE                  6    0.470  83.906   0.078   0.021   0.756   0.223
B C  2I9BE                  7    0.094  13.127  -0.812   0.022   0.552   0.426
E K  2I9BE                  8    0.633 130.270  -0.583   0.021   0.279   0.699
E T  2I9BE                  9    0.519  72.013  -0.893   0.018   0.088   0.893
E N  2I9BE                 10    0.555  81.267  -1.018   0.018   0.047   0.935
E G  2I9BE                 11    0.486  38.225  -0.979   0.018   0.047   0.935
E D  2I9BE                 12    0.571  82.209   0.692   0.004   0.138   0.858
B C  2I9BE                 13    0.078  10.993  -0.275   0.022   0.552   0.426
E R  2I9BE                 14    0.394  90.157   1.427   0.021   0.756   0.223
B V  2I9BE                 15    0.238  36.550   0.251   0.018   0.846   0.136
E E  2I9BE                 16    0.461  80.554   0.375   0.018   0.846   0.136
E E  2I9BE                 17    0.579 101.081   0.062   0.021   0.756   0.223
B C  2I9BE                 18    0.069   9.744  -0.283   0.022   0.359   0.619
E A  2I9BE                 19    0.420  46.339  -0.047   0.056   0.142   0.802
E L  2I9BE                 20    0.327  59.892  -0.648   0.053   0.043   0.903
E G  2I9BE                 21    0.402  31.630  -1.335   0.115   0.016   0.868
E Q  2I9BE                 22    0.400  71.476   0.515   0.181   0.016   0.803
E D  2I9BE                 23    0.482  69.399  -0.769   0.354   0.048   0.598
B L  2I9BE                 24    0.087  15.875   0.626   0.354   0.048   0.598
B C  2I9BE                 25    0.057   7.989   0.082   0.386   0.097   0.517
E R  2I9BE                 26    0.310  70.944   0.452   0.538   0.173   0.289
B T  2I9BE                 27    0.229  31.748  -0.336   0.453   0.248   0.299
B T  2I9BE                 28    0.159  22.039   0.593   0.478   0.309   0.214
B I  2I9BE                 29    0.101  18.685  -0.449   0.373   0.401   0.226
B V  2I9BE                 30    0.131  20.165   0.352   0.268   0.505   0.227
E R  2I9BE                 31    0.373  85.486   0.180   0.159   0.516   0.325
E L  2I9BE                 32    0.276  50.462   0.632   0.159   0.516   0.325
B W  2I9BE                 33    0.149  35.786   0.295   0.159   0.516   0.325
E E  2I9BE                 34    0.604 105.484   0.242   0.066   0.296   0.638
E E  2I9BE                 35    0.679 118.569  -0.346   0.052   0.084   0.864
E G  2I9BE                 36    0.432  34.014  -1.296   0.052   0.084   0.864
E E  2I9BE                 37    0.626 109.275  -0.029   0.056   0.142   0.802
E E  2I9BE                 38    0.483  84.433   0.536   0.021   0.451   0.528
B L  2I9BE                 39    0.218  39.879   0.613   0.023   0.655   0.322
E E  2I9BE                 40    0.462  80.781   0.125   0.021   0.756   0.223
B L  2I9BE                 41    0.243  44.457   0.474   0.018   0.846   0.136
B V  2I9BE                 42    0.212  32.646   0.159   0.011   0.918   0.071
E E  2I9BE                 43    0.498  87.088  -0.423   0.018   0.846   0.136
E K  2I9BE                 44    0.434  89.233   0.276   0.022   0.552   0.426
E S  2I9BE                 45    0.409  47.935  -1.566   0.066   0.296   0.638
B C  2I9BE                 46    0.134  18.870  -0.360   0.021   0.279   0.699
E T  2I9BE                 47    0.442  61.236  -0.836   0.022   0.359   0.619
E H  2I9BE                 48    0.313  56.971  -0.021   0.020   0.205   0.775
E S  2I9BE                 49    0.584  68.410  -0.574   0.018   0.088   0.893
E E  2I9BE                 50    0.736 128.544  -0.393   0.018   0.047   0.935
E K  2I9BE                 51    0.493 101.431  -0.211   0.052   0.084   0.864
E T  2I9BE                 52    0.486  67.450  -0.918   0.056   0.142   0.802
E N  2I9BE                 53    0.468  68.574  -1.124   0.052   0.084   0.864
E R  2I9BE                 54    0.362  82.990  -0.460   0.064   0.216   0.721
E T  2I9BE                 55    0.359  49.738  -0.729   0.069   0.386   0.545
B L  2I9BE                 56    0.180  32.903  -0.117   0.074   0.484   0.442
B S  2I9BE                 57    0.274  32.125  -0.869   0.079   0.592   0.329
B Y  2I9BE                 58    0.227  48.553  -0.547   0.169   0.612   0.219
E R  2I9BE                 59    0.325  74.471  -0.575   0.159   0.516   0.325
B T  2I9BE                 60    0.284  39.405  -0.832   0.135   0.317   0.548
B G  2I9BE                 61    0.294  23.122  -1.513   0.135   0.317   0.548
E L  2I9BE                 62    0.355  65.037  -1.175   0.148   0.418   0.435
E K  2I9BE                 63    0.386  79.318   0.051   0.087   0.683   0.230
B I  2I9BE                 64    0.175  32.375  -0.248   0.085   0.775   0.140
B T  2I9BE                 65    0.187  25.923   0.024   0.087   0.683   0.230
E S  2I9BE                 66    0.316  37.035  -0.378   0.169   0.612   0.219
B L  2I9BE                 67    0.146  26.696   0.009   0.148   0.418   0.435
E T  2I9BE                 68    0.378  52.415  -0.297   0.231   0.330   0.439
E E  2I9BE                 69    0.395  69.041  -0.617   0.231   0.330   0.439
B V  2I9BE                 70    0.234  35.935  -0.640   0.231   0.330   0.439
B V  2I9BE                 71    0.168  25.806  -0.207   0.252   0.423   0.325
B C  2I9BE                 72    0.123  17.283  -0.275   0.252   0.423   0.325
E G  2I9BE                 73    0.370  29.111  -1.410   0.125   0.227   0.648
B L  2I9BE                 74    0.262  47.972  -1.032   0.118   0.150   0.732
E D  2I9BE                 75    0.395  56.876  -1.299   0.113   0.087   0.800
B L  2I9BE                 76    0.162  29.662  -0.873   0.113   0.087   0.800
B C  2I9BE                 77    0.138  19.319  -1.297   0.053   0.043   0.903
E N  2I9BE                 78    0.437  63.904  -1.420   0.053   0.043   0.903
E Q  2I9BE                 79    0.507  90.586  -0.299   0.058   0.017   0.925
E G  2I9BE                 80    0.554  43.631  -1.582   0.018   0.019   0.964
E N  2I9BE                 81    0.584  85.527  -1.631   0.018   0.019   0.964
E S  2I9BE                 82    0.535  62.679  -1.921   0.018   0.019   0.964
E G  2I9BE                 83    0.394  30.968  -1.747   0.018   0.019   0.964
E R  2I9BE                 84    0.512 117.179  -1.040   0.018   0.047   0.935
E A  2I9BE                 85    0.546  60.202  -2.247   0.018   0.088   0.893
E V  2I9BE                 86    0.305  46.832  -0.220   0.019   0.141   0.840
E T  2I9BE                 87    0.479  66.479  -0.866   0.021   0.279   0.699
B Y  2I9BE                 88    0.125  26.606  -0.184   0.021   0.279   0.699
E S  2I9BE                 89    0.425  49.751  -1.266   0.019   0.141   0.840
E R  2I9BE                 90    0.536 122.744  -1.116   0.005   0.045   0.951
E S  2I9BE                 91    0.504  59.034  -1.749   0.004   0.085   0.910
B R  2I9BE                 92    0.275  62.975   0.810   0.005   0.262   0.733
E Y  2I9BE                 93    0.297  63.383  -0.839   0.004   0.616   0.381
B L  2I9BE                 94    0.055  10.034   0.331   0.002   0.816   0.182
B E  2I9BE                 95    0.260  45.335   0.227   0.001   0.900   0.099
B C  2I9BE                 96    0.038   5.265  -0.381   0.018   0.846   0.136
B I  2I9BE                 97    0.278  51.504  -0.229   0.018   0.846   0.136
E S  2I9BE                 98    0.354  41.536  -0.368   0.018   0.846   0.136
B C  2I9BE                 99    0.075  10.488  -0.434   0.021   0.451   0.528
E G  2I9BE                100    0.442  34.809  -0.429   0.019   0.141   0.840
E S  2I9BE                101    0.517  60.592  -1.061   0.004   0.085   0.910
E S  2I9BE                102    0.559  65.468  -0.869   0.018   0.047   0.935
E D  2I9BE                103    0.618  88.996  -2.170   0.018   0.019   0.964
E M  2I9BE                104    0.488  97.589  -0.817   0.005   0.015   0.979
E S  2I9BE                105    0.572  67.062   0.057   0.004   0.085   0.910
B C  2I9BE                106    0.140  19.712  -0.142   0.004   0.197   0.799
E H  2I9BE                107    0.629 114.470   0.080   0.004   0.420   0.576
E Q  2I9BE                108    0.486  86.782   0.103   0.004   0.616   0.381
E S  2I9BE                109    0.575  67.402   0.628   0.003   0.718   0.279
B L  2I9BE                110    0.209  38.323   0.909   0.003   0.718   0.279
E Q  2I9BE                111    0.688 122.930   0.823   0.004   0.616   0.381
B C  2I9BE                112    0.055   7.792  -0.806   0.004   0.197   0.799
E R  2I9BE                113    0.593 135.889  -0.271   0.005   0.045   0.951
E S  2I9BE                114    0.497  58.272  -0.550   0.018   0.019   0.964
E P  2I9BE                115    0.587  83.366  -0.588   0.016   0.005   0.979
E E  2I9BE                116    0.522  91.141   0.117   0.018   0.019   0.964
E E  2I9BE                117    0.548  95.788   0.069   0.019   0.141   0.840
E Q  2I9BE                118    0.329  58.795   0.572   0.004   0.420   0.576
B C  2I9BE                119    0.088  12.313  -0.224   0.003   0.718   0.279
B L  2I9BE                120    0.198  36.217   0.818   0.011   0.918   0.071
E D  2I9BE                121    0.364  52.409  -0.282   0.011   0.918   0.071
B V  2I9BE                122    0.173  26.575   0.378   0.011   0.918   0.071
B V  2I9BE                123    0.127  19.474   0.532   0.006   0.962   0.032
B T  2I9BE                124    0.151  20.999   0.228   0.011   0.918   0.071
B H  2I9BE                125    0.216  39.290   0.294   0.011   0.918   0.071
E W  2I9BE                126    0.296  71.284   0.157   0.018   0.846   0.136
B I  2I9BE                127    0.193  35.705  -0.810   0.022   0.552   0.426
E Q  2I9BE                128    0.529  94.426  -1.369   0.064   0.216   0.721
E R  2I9BE                129    0.442 101.195  -0.172   0.019   0.141   0.840
E P  2I9BE                130    0.444  63.004  -1.543   0.018   0.088   0.893
E K  2I9BE                131    0.545 112.086  -1.222   0.018   0.088   0.893
E D  2I9BE                132    0.443  63.836  -1.714   0.019   0.141   0.840
E D  2I9BE                133    0.527  75.883  -0.977   0.019   0.141   0.840
E R  2I9BE                134    0.470 107.722  -0.740   0.021   0.279   0.699
E H  2I9BE                135    0.317  57.571  -0.294   0.021   0.451   0.528
B L  2I9BE                136    0.220  40.282  -1.253   0.022   0.552   0.426
E R  2I9BE                137    0.353  80.745  -0.256   0.021   0.451   0.528
B G  2I9BE                138    0.319  25.105  -2.528   0.022   0.359   0.619
B C  2I9BE                139    0.125  17.578  -0.591   0.020   0.205   0.775
B G  2I9BE                140    0.256  20.131  -1.925   0.020   0.205   0.775
E Y  2I9BE                141    0.410  87.510  -1.107   0.018   0.088   0.893
E L  2I9BE                142    0.383  70.127  -1.590   0.018   0.019   0.964
E P  2I9BE                143    0.664  94.278  -1.767   0.018   0.019   0.964
E G  2I9BE                144    0.569  44.757  -1.743   0.003   0.003   0.994
B C  2I9BE                145    0.078  11.007  -0.834   0.018   0.047   0.935
E P  2I9BE                146    0.374  53.042  -1.177   0.019   0.141   0.840
B G  2I9BE                147    0.168  13.214  -2.217   0.004   0.197   0.799
E S  2I9BE                148    0.351  41.079  -1.271   0.021   0.279   0.699
B N  2I9BE                149    0.195  28.577  -1.658   0.019   0.141   0.840
E G  2I9BE                150    0.372  29.284  -1.637   0.018   0.088   0.893
B F  2I9BE                151    0.243  48.750  -1.487   0.053   0.043   0.903
E H  2I9BE                152    0.341  61.973  -1.795   0.113   0.043   0.844
E N  2I9BE                153    0.356  52.060  -0.911   0.191   0.086   0.723
B Q  2I9BE                154    0.288  51.365  -0.926   0.307   0.165   0.527
B D  2I9BE                155    0.259  37.351   0.251   0.231   0.330   0.439
B T  2I9BE                156    0.100  13.925  -0.347   0.268   0.505   0.227
B F  2I9BE                157    0.136  27.335   0.139   0.273   0.587   0.140
B H  2I9BE                158    0.286  52.060  -0.175   0.273   0.587   0.140
B F  2I9BE                159    0.239  48.048  -0.431   0.252   0.423   0.325
E L  2I9BE                160    0.286  52.422   0.354   0.231   0.330   0.439
E K  2I9BE                161    0.501 103.076   1.202   0.216   0.235   0.548
B C  2I9BE                162    0.096  13.492   0.019   0.199   0.152   0.649
B C  2I9BE                163    0.082  11.555   0.180   0.199   0.152   0.649
E Q  2I9BE                164    0.495  88.461   0.242   0.113   0.087   0.800
E T  2I9BE                165    0.517  71.708  -0.147   0.184   0.043   0.773
E T  2I9BE                166    0.557  77.214  -0.435   0.113   0.043   0.844
E K  2I9BE                167    0.473  97.358   0.843   0.113   0.043   0.844
B C  2I9BE                168    0.078  10.951  -1.533   0.113   0.043   0.844
E N  2I9BE                169    0.446  65.368  -0.526   0.053   0.043   0.903
E E  2I9BE                170    0.673 117.486  -0.752   0.053   0.043   0.903
E G  2I9BE                171    0.605  47.590  -0.842   0.053   0.043   0.903
E P  2I9BE                172    0.514  72.894  -0.929   0.053   0.043   0.903
B I  2I9BE                173    0.267  49.377  -0.941   0.058   0.017   0.925
E L  2I9BE                174    0.331  60.679  -1.171   0.058   0.017   0.925
E E  2I9BE                175    0.599 104.575  -0.128   0.058   0.017   0.925
E L  2I9BE                176    0.411  75.236  -1.767   0.058   0.017   0.925
E E  2I9BE                177    0.676 118.185  -1.196   0.058   0.017   0.925
E N  2I9BE                178    0.564  82.496  -1.794   0.058   0.017   0.925
E L  2I9BE                179    0.392  71.702  -0.628   0.018   0.019   0.964
E P  2I9BE                180    0.437  62.010  -0.661   0.018   0.019   0.964
E Q  2I9BE                181    0.496  88.532  -1.199   0.053   0.043   0.903
E N  2I9BE                182    0.388  56.774  -1.496   0.018   0.047   0.935
E G  2I9BE                183    0.354  27.883  -1.700   0.018   0.047   0.935
E R  2I9BE                184    0.356  81.547  -1.215   0.019   0.141   0.840
E Q  2I9BE                185    0.421  75.226  -0.235   0.022   0.359   0.619
B C  2I9BE                186    0.064   8.986  -0.193   0.022   0.359   0.619
B Y  2I9BE                187    0.244  52.164  -0.138   0.022   0.359   0.619
B S  2I9BE                188    0.245  28.714  -1.341   0.022   0.359   0.619
B C  2I9BE                189    0.103  14.503  -0.757   0.022   0.359   0.619
E K  2I9BE                190    0.424  87.196  -0.011   0.069   0.386   0.545
E G  2I9BE                191    0.364  28.623  -2.360   0.064   0.216   0.721
E Q  2I9BE                192    0.472  84.335  -1.510   0.064   0.216   0.721
E S  2I9BE                193    0.437  51.263  -1.255   0.064   0.216   0.721
E T  2I9BE                194    0.489  67.838  -0.673   0.064   0.216   0.721
E H  2I9BE                195    0.506  92.023  -0.567   0.064   0.216   0.721
E G  2I9BE                196    0.531  41.790   0.290   0.066   0.296   0.638
B C  2I9BE                197    0.179  25.160  -1.701   0.066   0.296   0.638
E S  2I9BE                198    0.437  51.240  -0.825   0.056   0.142   0.802
E S  2I9BE                199    0.542  63.546  -1.752   0.052   0.084   0.864
E E  2I9BE                200    0.601 104.925  -0.308   0.052   0.084   0.864
E E  2I9BE                201    0.367  64.167  -0.437   0.019   0.141   0.840
E T  2I9BE                202    0.374  51.888  -0.824   0.022   0.359   0.619
E F  2I9BE                203    0.324  65.007  -0.662   0.022   0.552   0.426
E L  2I9BE                204    0.445  81.534  -0.147   0.023   0.655   0.322
B I  2I9BE                205    0.142  26.214  -0.039   0.021   0.756   0.223
E D  2I9BE                206    0.448  64.557  -0.482   0.023   0.655   0.322
B C  2I9BE                207    0.078  10.951  -2.205   0.021   0.451   0.528
E R  2I9BE                208    0.517 118.485  -0.241   0.021   0.279   0.699
E G  2I9BE                209    0.393  30.953  -1.364   0.018   0.088   0.893
E P  2I9BE                210    0.454  64.465  -1.011   0.018   0.019   0.964
E M  2I9BE                211    0.504 100.810  -1.265   0.058   0.017   0.925
E N  2I9BE                212    0.507  74.283  -0.319   0.053   0.043   0.903
B Q  2I9BE                213    0.240  42.935   1.179   0.020   0.205   0.775
B C  2I9BE                214    0.055   7.736   0.151   0.023   0.655   0.322
B L  2I9BE                215    0.088  16.076   0.499   0.018   0.846   0.136
E V  2I9BE                216    0.310  47.678   0.243   0.011   0.918   0.071
B A  2I9BE                217    0.057   6.270  -0.138   0.018   0.846   0.136
B T  2I9BE                218    0.196  27.116  -0.584   0.018   0.846   0.136
B G  2I9BE                219    0.151  11.923  -1.044   0.002   0.816   0.182
E T  2I9BE                220    0.286  39.627  -0.294   0.018   0.846   0.136
B H  2I9BE                221    0.248  45.202  -0.479   0.023   0.655   0.322
E E  2I9BE                222    0.406  70.981  -0.360   0.021   0.451   0.528
E P  2I9BE                223    0.445  63.075  -1.408   0.021   0.279   0.699
E K  2I9BE                224    0.519 106.799  -1.821   0.018   0.047   0.935
E Q  2I9BE                225    0.605 108.053  -1.803   0.018   0.047   0.935
E Q  2I9BE                226    0.383  68.386  -0.170   0.022   0.359   0.619
E S  2I9BE                227    0.441  51.709  -0.180   0.003   0.718   0.279
B Y  2I9BE                228    0.151  32.354   0.577   0.018   0.846   0.136
B M  2I9BE                229    0.196  39.120  -0.579   0.011   0.918   0.071
B V  2I9BE                230    0.087  13.341  -0.752   0.018   0.846   0.136
E R  2I9BE                231    0.269  61.647   0.145   0.021   0.756   0.223
B G  2I9BE                232    0.107   8.429  -1.792   0.022   0.552   0.426
B C  2I9BE                233    0.055   7.736  -0.053   0.074   0.484   0.442
B A  2I9BE                234    0.085   9.323  -0.372   0.135   0.317   0.548
B T  2I9BE                235    0.236  32.733   0.303   0.125   0.227   0.648
E A  2I9BE                236    0.510  56.180  -0.018   0.199   0.152   0.649
B S  2I9BE                237    0.322  37.738  -1.314   0.216   0.235   0.548
B A  2I9BE                238    0.274  30.140  -0.065   0.252   0.423   0.325
B H  2I9BE                239    0.099  17.972   0.228   0.159   0.516   0.325
E L  2I9BE                240    0.309  56.578   0.141   0.148   0.418   0.435
B G  2I9BE                241    0.244  19.234  -0.910   0.135   0.317   0.548
E D  2I9BE                242    0.413  59.513  -0.768   0.135   0.317   0.548
B A  2I9BE                243    0.240  26.470  -1.949   0.125   0.227   0.648
E F  2I9BE                244    0.365  73.336  -1.694   0.064   0.216   0.721
E S  2I9BE                245    0.376  44.009  -0.628   0.056   0.142   0.802
E M  2I9BE                246    0.429  85.923  -1.233   0.018   0.088   0.893
E N  2I9BE                247    0.459  67.227  -1.045   0.018   0.047   0.935
B H  2I9BE                248    0.387  70.413  -1.484   0.020   0.205   0.775
B I  2I9BE                249    0.163  30.081   0.007   0.004   0.420   0.576
E D  2I9BE                250    0.355  51.170  -0.882   0.004   0.616   0.381
B V  2I9BE                251    0.071  10.959   0.048   0.021   0.756   0.223
B S  2I9BE                252    0.185  21.647  -1.133   0.021   0.756   0.223
B C  2I9BE                253    0.051   7.146   0.156   0.022   0.552   0.426
B C  2I9BE                254    0.069   9.730  -0.291   0.021   0.451   0.528
E T  2I9BE                255    0.544  75.453  -0.139   0.019   0.141   0.840
E K  2I9BE                256    0.520 107.005  -1.792   0.018   0.019   0.964
E S  2I9BE                257    0.568  66.605  -1.821   0.018   0.019   0.964
E G  2I9BE                258    0.381  30.016  -0.935   0.018   0.019   0.964
B C  2I9BE                259    0.111  15.542  -1.585   0.018   0.019   0.964
E N  2I9BE                260    0.418  61.254  -1.349   0.018   0.019   0.964
E H  2I9BE                261    0.417  75.870   0.082   0.018   0.019   0.964
E P  2I9BE                262    0.468  66.423  -0.792   0.018   0.019   0.964
E D  2I9BE                263    0.647  93.262  -1.587   0.018   0.019   0.964
E L  2I9BE                264    0.521  95.358  -0.583   0.018   0.019   0.964
E D  2I9BE                265    0.921 132.788  -0.361   0.003   0.003   0.994

Explain the output.  Go back.
```

---
